# Validation of the cell cycle progression score to differentiate indolent from aggressive prostate cancer in men diagnosed through transurethral resection of the prostate biopsy

**DOI:** 10.1002/cnr2.1535

**Published:** 2021-08-22

**Authors:** Jack M. Cuzick, Steven Stone, Lauren Lenz, Darl D. Flake, Saradha Rajamani, Henrik Moller, Daniel Maurice Berney, Todd Cohen, Peter T. Scardino

**Affiliations:** ^1^ Centre for Cancer Prevention, Wolfson Institute of Preventive Medicine Queen Mary University of London London UK; ^2^ Myriad Genetics, Inc. Salt Lake City Utah USA; ^3^ Department of Cancer Epidemiology, Population and Global Health King's College London London UK; ^4^ Department of Molecular Oncology, Barts Cancer Institute Queen Mary University of London London UK; ^5^ Department of Urology Memorial Sloan‐Kettering Cancer Center New York New York USA

**Keywords:** cancer prevention, cell cycle, epidemiology and prevention, prostate cancer

## Abstract

**Background:**

Validation of biomarker‐based prognostic models to improve risk stratification in men with localized prostate cancer (PrCa) remains a clinical need. It has previously been shown that the cell cycle progression (CCP) test provides significant, independent prognostic information for men who were incidentally diagnosed with PrCa after transurethral resection of the prostate (TURP) and were conservatively managed.

**Aim:**

The results have been extended in a newly analyzed retrospective cohort of UK men diagnosed through TURP biopsy (TURP1B; *N* = 305).

**Methods and Results:**

The CCP score was derived from TURP biopsy tissue and combined with a modified UCSF Cancer of the Prostate Risk Assessment score (CAPRA) to generate the clinical cell‐cycle risk score (CCR). The primary endpoint was PrCa‐specific mortality (PSM). Hazard ratios (HR) were calculated for a one‐unit change in score. Median follow‐up was 9.6 (IQR: 5.4, 14.1) years, and 67 (22%) men died from PrCa within 10 years of diagnosis. The median CCP score was 1.1 (IQR: 0.6, 1.7). In univariate analyses, CCR proved a significant prognosticator of PSM (HR per unit score change = 2.28; 95% CI: 1.89, 2.74; *P* = 1.0 × 10^−19^). In multivariate analyses, CCR remained a significant prognosticator of PSM after adjusting for CAPRA (HR per unit score change = 4.36; 95% CI: 2.65, 7.16; *P* = 1.3 × 10^−8^), indicating that its molecular component, CCP, provides significant, independent prognostic information.

**Conclusion:**

These findings validate a combined clinicopathologic and molecular prognostic model for conservatively managed men who are diagnosed through TURP, supporting the use of CCR to inform clinical management.

## 1 INTRODUCTION

Increasingly, clinical management of cancer patients is being tailored to the individual's risk of having or developing aggressive disease. This is particularly important for patients with newly diagnosed prostate cancer (PrCa), wherein treatment options range from conservative management with active surveillance or watchful waiting, to definitive treatment such as prostatectomy or primary radiation with or without concurrent androgen deprivation therapy (ADT).

For patients with low‐risk disease, conservative management is becoming increasingly popular. A recent survey of clinical practice in the United States found that approximately 40% of men with low‐risk clinical features are opting for active surveillance.^1^ For these patients, deferred treatment can be more appropriate because the risk of disease progression is low, and it offers the opportunity to avoid unnecessary treatment‐associated morbidities such as impotence and incontinence. Conversely, when high risk can be established, definitive treatment is appropriate. Randomized clinical trial results^2^, ^3^ and clinical practice guidelines^4^, ^5^, ^6^, ^7^ both support the use of either surgery and adjuvant radiation, or primary radiation and ADT in appropriately selected patients.

However, there are potential risks associated with any clinical management decision. The opportunity to cure localized disease may be lost for patients who are inappropriately managed by AS, or, conversely, patients who are inaccurately identified as high risk may suffer needlessly from treatment‐associated morbidities. Therefore, accurate risk estimates are essential. Various prognostic models have been developed and validated to help predict disease outcome. For example, the UCSF Cancer of the Prostate Risk Assessment (CAPRA) score combines Gleason score, prostate‐specific antigen (PSA) level, clinical T Stage, percent positive cores, and age into a validated prognostic model for biochemical recurrence (BCR) after definitive treatment and for prostate cancer‐specific mortality (PSM) after conservative management.^8^, ^9^ Another prognostic model, PREDICT, uses similar variables as CAPRA to predict distal oncologic outcomes but also estimates the absolute benefit of treatment.^10^ While these are important tools for helping physicians and patients make clinical management decisions, better discrimination is needed, and this is especially true for men diagnosed through TURP, since most prognostic models were developed and validated primarily for men diagnosed with needle biopsy.

Numerous studies have demonstrated unequivocally that tumor‐derived molecular prognostic information improves risk stratification for newly diagnosed PrCa patients, compared with clinicopathologic features alone.^11^ In addition, the National Comprehensive Cancer Network, the American Society for Clinical Oncology, and the European Association for Urology have recognized the importance of molecular information for improving risk stratification.^4^, ^5^, ^6^, ^7^ Thus, molecular tests have emerged as an important clinical tool to augment clinical variables in guiding appropriate clinical management. The cell cycle progression (CCP) gene expression classifier test (Prolaris) is a validated molecular test based on measuring the expression levels of 31 genes involved in cell proliferation. The CCP test has been evaluated in numerous clinical settings and shown to add independent prognostic information that is not captured by standard clinicopathologic features.^12^, ^13^, ^14^, ^15^ The score has been combined with clinical variables into a combined clinical cell‐cycle risk score (CCR) to calculate a 10‐year predicted risk of PSM.^12^

The objective of this study was to validate a predefined model (CCR) that combines molecular and clinicopathologic variables to predict risk of aggressive disease in a retrospective cohort of men who were incidentally diagnosed with PrCa by TURP biopsy and were conservatively managed. These patients were collected as part of a TURP cohort, part of which was used previously to evaluate the molecular CCP score,^16^ but the patients used in the present analysis were not included in the previous analysis and the CCR score which combines CCP with CAPRA^12^ was not evaluated in the previous analysis. We also aimed to expand the body of evidence showing that CCP provides significant prognostic information that is not captured by clinicopathologic features.

## 2 MATERIALS AND METHODS

### 2.1 Cohort

The TURP1B cohort was part of a large, previously described cohort of conservatively managed men, but this subgroup has not been analyzed previously. The full TURP cohort was a population‐based, retrospective, watchful‐waiting cohort identified from six cancer registries in Great Britain. Within each region, cases from collaborating hospitals were reviewed; full details of these cases have been reported.^16^ Men were included in the study if they had clinically localized PrCa diagnosed by TURP from 1990 through 1996, were younger than 76 years at diagnosis, and had a baseline PSA measurement <100 ng/mL. Patients were excluded if they were treated with radical prostatectomy or radiation therapy, or if they died, or showed evidence of metastatic disease within 6 months of diagnosis. Men who had hormone therapy before diagnostic biopsy were also excluded due to the potential effects of hormone treatment on Gleason score interpretation. Original histological specimens for difficult cases were centrally reviewed by a panel of expert urological pathologists to confirm the TURP diagnosis and, when necessary, to reassign Gleason scores for all tumors using a contemporary interpretation of the Gleason scoring system.^17^ Follow‐up information was obtained through the cancer registries, with the final review conducted in January 2010. Deaths were divided into two categories, those from PrCa and those from other causes, according to the World Health Organization's standardized criteria.^18^

**FIGURE 1:**
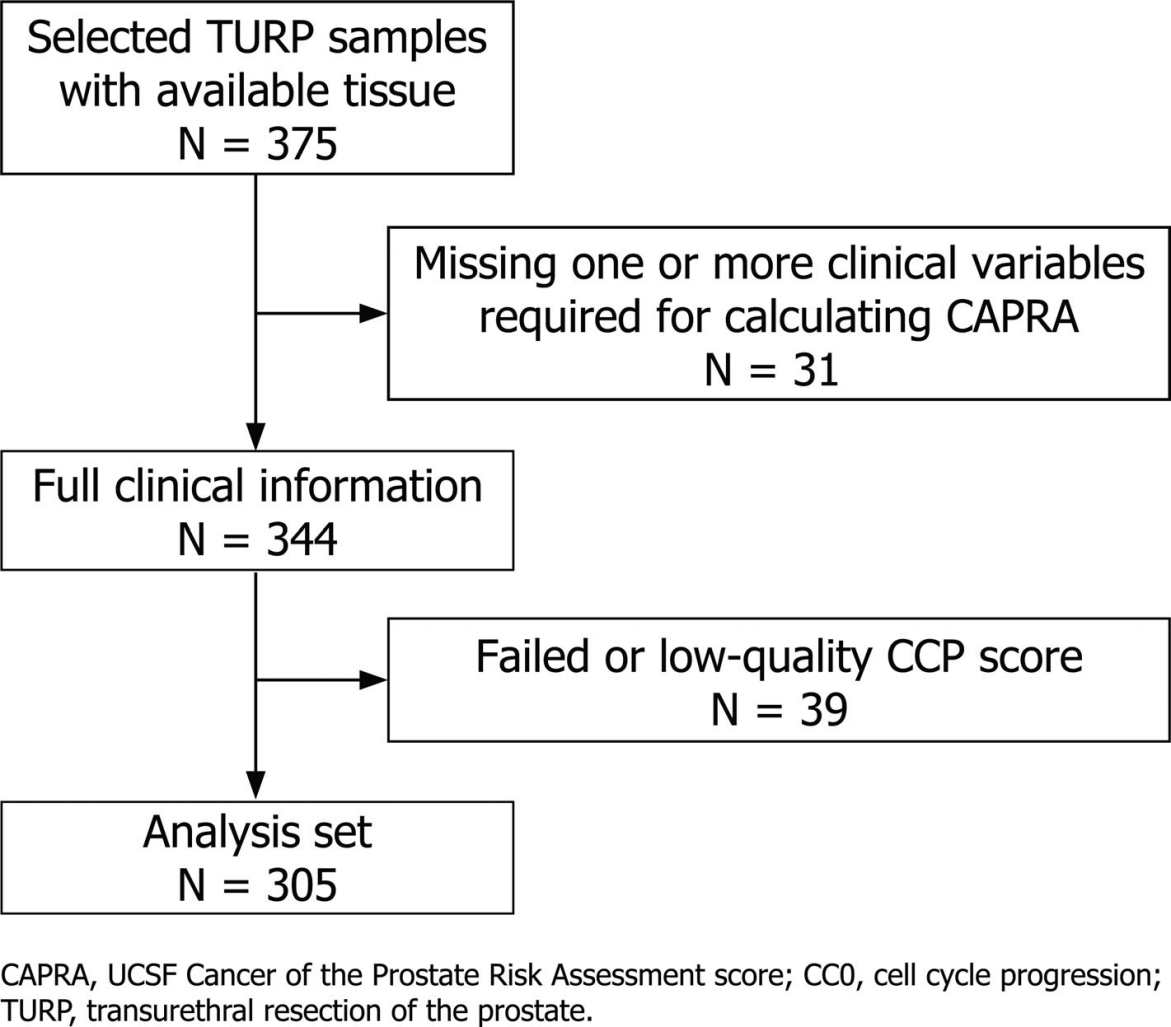
Patient flow for TURP1B

A sub‐cohort of a large cohort of conservatively managed men who were diagnosed by TURP biopsy has been previously reported.^16^ The sub‐cohort analyzed here, TURP1B, is an independent subset of the full cohort which has not been previously analyzed. This new cohort comprised 305 men who were eligible for the study, generated a valid CCP score, and had a complete set of clinicopathologic features to allow calculation of a CAPRA score (Figure 1). A previous independent TURP sub‐cohort (TURP1A) was collected at the same time as TURP1B, and has been reported previously^16^ and was also used to help develop the CCR score.^12^ TURP1A comprised 330 men who met the same eligibility criteria as for TURP1B.

### 2.2 CCP and CCR score

CCP testing was performed on the diagnostic TURP biopsy tissue. Depending on tumor volume, 5–12 consecutive, 5 μm formalin‐fixed, paraffin‐embedded tumor sections were used to isolate RNA. The tumor region was macro dissected from the slide according to the pathologist's instructions. Molecular testing was performed (blinded per author guidelines). Briefly, RNA extraction was performed using miRNeasy (Qiagen, Hilden, Germany). The expression of 31 CCP genes and 15 housekeeper genes was quantified in triplicate using TaqMan Low Density Arrays (Applied Biosystems, Foster City, CA). The CCP score was calculated as the average expression of the CCP genes normalized by the expression of the housekeeper genes. After measuring RNA expression levels, the CCP molecular score was combined with the CAPRA score to produce the predefined CCR score [(0.39 × CAPRA) + (0.57 × CCP)].^12^

### 2.3 Calculation of CAPRA

In addition to age, Gleason score, PSA and clinical T stage, calculation of CAPRA requires percent positive cores (i.e., the number of needle cores containing cancer out of the number of needle cores taken at biopsy).^19^, ^20^ For patients diagnosed by TURP biopsy, we have replaced this with percent of positive TURP tissue chips, using the same weighting for different percentage categories as in the original CAPRA algorithm. As per definition of clinical stage, patients diagnosed through TURP and missing stage information were assumed to be T1.

### 2.4 Statistical analysis

For the primary endpoint, time to PrCa specific mortality, patients with events or last follow‐up after 10 years were censored at 10 years. Hazard ratios (HR) with 95% profile likelihood‐based confidence intervals and two‐sided *P*‐values from partial likelihood ratio tests were reported for all Cox proportional hazards models. Confidence intervals for risk estimates were based on the log–log transformation. After examining the Schoenfeld residuals, time dependence of predictors in Cox proportional hazards models was accounted for using untransformed time. The distribution of continuous variables was compared using the Wilcoxon rank‐sum test, and the distribution of ordered categorical variables was compared using the Goodman–Kruskal test. Calibration was tested using the Greenwood‐Nam‐D'Agostino test. *P*‐values were considered significant at the two‐sided 0.05 level. All analyses were carried out using R software, version 3.5.0 or higher (R Core Team, Vienna, Austria).

## 3 RESULTS

Median follow‐up for TURP1B was 9.6 years (IQR 5.4, 14.1), and 67 (22%) men died from PrCa within 10 years of diagnosis. The median CCP score was 1.1 (IQR: 0.6, 1.7). Compared with other clinicopathologic features, the CCP score correlated most strongly with Gleason score (Pearson *r* = 0.55). Median follow‐up for TURP1A was 9.8 years (IQR: 4.9, 11.6), and 67 (20%) men died from PrCa within 10‐years of disease diagnosis. The demographic features of TURP1A and TURP1B were similar except for a shift to higher clinical stage in TURP1B (Table 1).

**TABLE 1:** Comparison of the characteristics of the TURP1B cohort compared with those from the TURP1A cohort

	TURP1A (N = 330)	TURP1B (N = 305)	*P* Value
	Median (IQR)	Median (IQR)	
Age	71 (67, 73)	71 (67, 74)	.52
PSA	8.2 (2.7, 21.0)	9.6 (2.9, 25.3)	.15
CCR	1.57 (0.73, 3.02)	2.18 (1.07, 3.47)	3.5 × 10^−4^
CCP	0.7 (0.1, 1.3)	1.1 (0.6, 1.7)	7.6 × 10^−10^
CAPRA	3 (1, 6)	4 (1, 7)	.093
CAPRA risk category	*N* (%)	*N* (%)	–
Low risk (0–2)	141 (42.7%)	121 (39.7%)	.12
Intermediate risk (3–5)	97 (29.4%)	75 (24.6%)	
High risk (6–10)	92 (27.9%)	109 (35.7%)	
Clinical T stage	N(%)	N(%)	–
T1	239 (72.4%)	188 (61.6%)	.002
T2	63 (19.0%)	73 (23.9%)	
T3	28 (8.5%)	44 (14.4%)	
Extent of cancer (PPC surrogate)	15.0% (5.0%, 57.8%)	20.5% (6.1%, 52.8%)	.29
Gleason score	*N* (%)	*N* (%)	–
<7	169 (51.2%)	145 (47.5%)	.92
7	72 (21.8%)	92 (30.2%)	
>7	89 (27.0%)	68 (22.3%)	

*Note*: The distribution of continuous variables was compared using the Wilcoxon rank‐sum test, and the distribution of ordered categorical variables was compared using the Goodman–Kruskal test.

Abbreviations: CAPRA, UCSF Cancer of the Prostate Risk Assessment score; CCP, cell cycle progression score; CCR, clinical cell‐cycle risk score; IQR, interquartile ratio; PPC, percent of positive TURP chips; PSA, prostate‐specific antigen.

**FIGURE 2:**
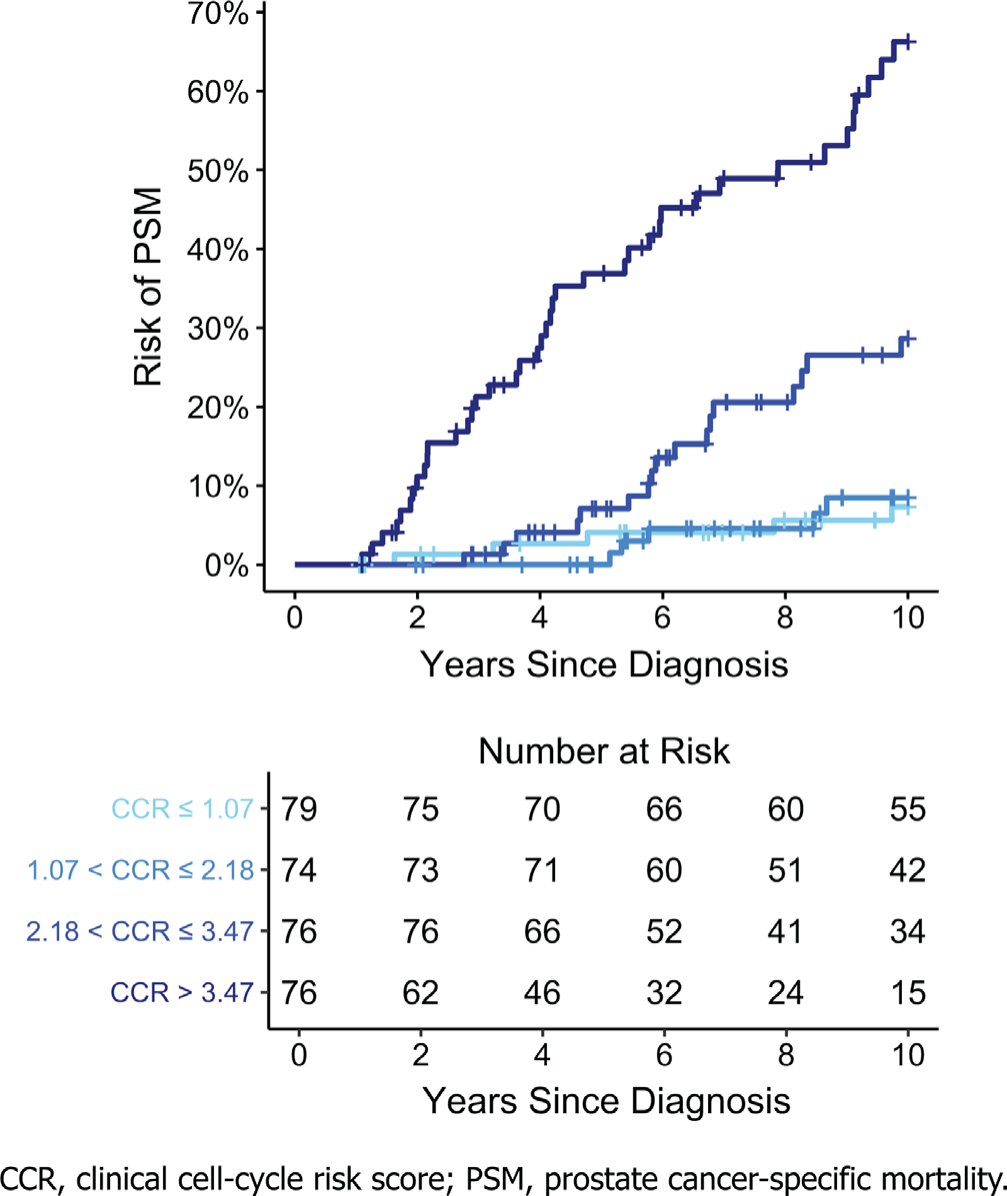
Kaplan–Meier risk curves for prostate cancer‐specific mortality, by clinical cell‐cycle risk score quartiles

**FIGURE 3:**
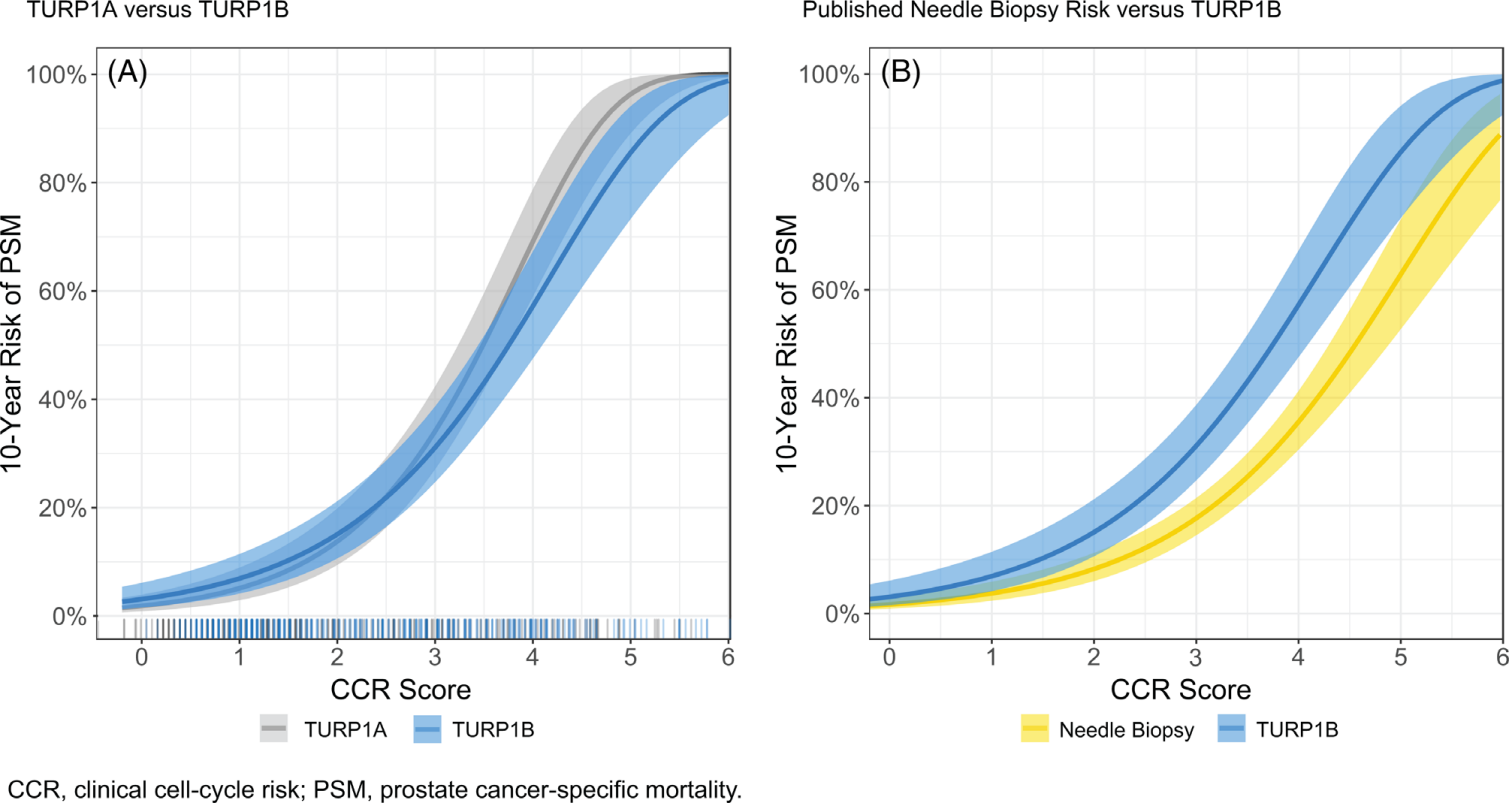
Clinical cell‐cycle risk score‐based risk curve for TURP1B compared with (A) TURP1A and (B) previously published risk data for men diagnosed using needle biopsy

In univariate analyses, the CCP score was a significant prognostic variable for PSM in the TURP1B cohort, with an HR per unit score change of 3.23 (95% CI: 2.50, 4.17, *P* = 1.9 × 10^−16^; Table 2). The CCR score was also a significant univariate prognosticator of PSM in the TURP1B cohort, with an HR per unit score change of 2.28 (95%CI: 1.89, 2.74, *P* = 1.0 × 10^−19^; Table 2). There was no evidence for time dependence within this model indicating that the prognostic value of CCR was similar across all time points (test using Schoenfeld residuals *P* = 0.13). The 10‐year Kaplan–Meier estimate of the probability of progression to PSM separated by CCR quartile groups in the TURP1B cohort is illustrated in Figure 2. The CCR score was used to generate a risk curve for progression to PSM within 10 years (Figure 3) and compared with the CCR‐based risk curve generated from TURP1A (Figure 3(A)). Although the curves were similar, there was evidence of overfitting in TURP1A, which had been used in part to train the CCR score.^12^ Risk predicted from the TURP1A model was also not significantly different from the observed risk in TURP1B, indicating good calibration (*P* = 0.28, Supplementary Figure). We also compared the CCR risk curve from TURP1B to that generated and validated for a separate cohort of patients diagnosed with needle biopsy (Figure 3(B)).^9^ Interestingly, men diagnosed with TURP had a higher risk of harboring aggressive disease compared with that among men who were diagnosed with needle biopsy, but had otherwise similar clinicopathologic and molecular prognostic features.

**TABLE 2:** Univariate and bivariate analysis of the TURP 1B cohort

	Univariate				CAPRA + CCR				
	HR per unit change (95% CI)	HR per SD (95% CI)	*χ* ^2^	*P*	HR per unit change^a^ (95% CI)	HR per *SD* ^b^ (95% CI)	Δ*χ* ^2^	*P*
CCP	3.23 (2.50, 4.17)	2.70 (2.17, 3.35)	67.7	1.9 × 10^−16^	–	–	–	–
CAPRA	1.38 (1.26, 1.51)	2.57 (1.99, 3.32)	57.7	3.1 × 10^−14^	0.71 (0.55, 0.91)	0.36 (0.17, 0.75)	7.5	0.0063
CCR	2.28 (1.89, 2.74)	3.26 (2.50, 4.25)	82.6	1.0 × 10^−19^	4.36 (2.65, 7.16)	8.27 (4.06, 16.84)	32.4	1.3 × 10^−8^

*Note*: The *χ* ^2^ values for the bivariate model are for the added information by the given variable when the other is already included.

Abbreviations: 95% CI, 95% confidence interval; CAPRA, UCSF Cancer of the Prostate Risk Assessment score; CCP, cell cycle progression score; CCR, clinical cell‐cycle risk score; df, degrees of freedom; HR, hazard ratio; SD, standard deviation.

^a^ Taken from bivariate model.

^b^ 2 df *χ* ^2^ for CCR + CAPRA = 90.1.

**FIGURE 4:**
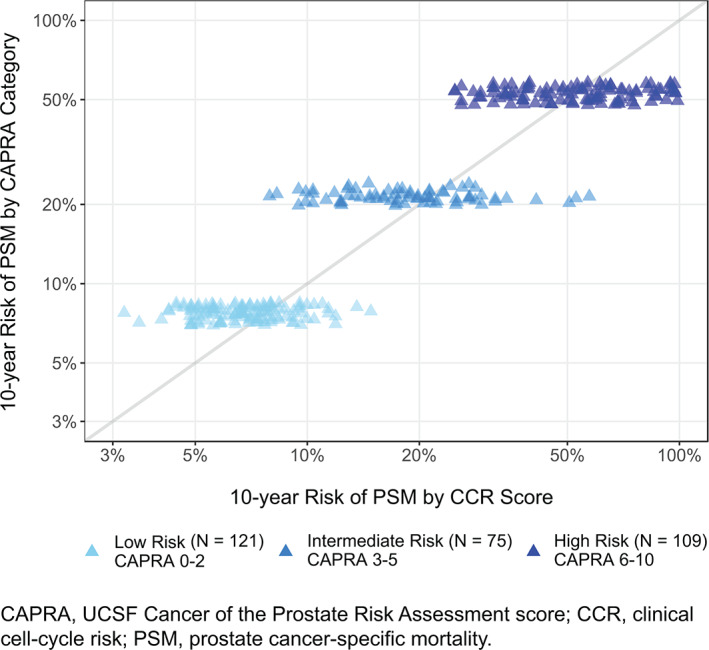
Comparison of 10‐year clinical cell‐cycle risk score across CAPRA (UCSF Cancer of the Prostate Risk Assessment) groups prostate cancer‐specific mortality risk estimates

In multivariate analyses (Table 2), the CCR score remained a significant prognostic factor of PSM after adjusting for CAPRA (HR per unit score = 4.36 (95% CI: 2.65, 7.16, *P* = 1.3 × 10^−8^), indicating that the molecular component of CCR provides significant and independent prognostic information. CAPRA was also significant in this analysis (*P* = 0.0063), but the HR was less than 1. This suggests that CAPRA is over‐weighted within the CCR score when evaluated by the TURP1B cohort. The amount of new prognostic information provided by the CCR score can be illustrated by comparing the difference in predicted risk between CCR‐only and CAPRA‐only prognostic models (Figure 4). In addition, the c‐index for the CAPRA‐only model was 0.76 and improved to 0.80 for CCR.

## 4 DISCUSSION

With increasing frequency, men with low‐risk PrCa are choosing conservative clinical management regimens such as active surveillance. Validated biomarkers that add independent risk discrimination are useful adjuncts for identifying suitable candidates for such management. They can also be used to help guide the intensity of invention if immediate treatment is deemed necessary. Here, we have provided additional evidence that the CCP score provides a significant and substantial amount of independent prognostic information in men who were incidentally diagnosed with PrCa after TURP and conservatively managed. We have also validated a predefined prognostic model (the CCR score) that combines molecular and clinicopathologic data to predict the 10‐year risk of PSM.

The CCR score was trained in more than 1000 men,^12^ including a previous cohort of men diagnosed with TURP biopsy.^16^ It was subsequently validated in numerous clinical settings, including both conservative management and immediate treatment,^12^, ^13^, ^14^, ^15^, ^21^ but had not been validated in men diagnosed through TURP. In this study, we evaluated CCR in an independent cohort of men who were incidentally diagnosed with PrCa after TURP biopsy. There is evidence that the combined CCR score is not optimal for TURP1B patients. In a bivariable model that included CAPRA and CCR, CAPRA remained significant but with an HR of 0.71 (Table 2). This suggests that CCR over‐weights the clinical variables, at least when applied to the TURP1B cohort. However, in the bivariable model, the increase in *χ* ^2^ value for adding CCR to CAPRA (*χ* ^2^ = 32.4) is substantially larger than that for adding CAPRA to CCR (*χ* ^2^ = 7.5). Therefore, the relevant amount of over‐weighting is small compared with the amount of prognostic information captured by CCR, and it would have a minor impact on estimated risk. It is also true that univariate analysis indicates that the CCR score provides substantially more prognostic information than is captured by the CAPRA‐only model, indicating that there is significant information to be gained from the molecular portion of the CCR score. Finally, it should be noted that TURP1B is a relatively small cohort, and therefore spurious cohort specific results on prognostic models that are not valid or reproducible are possible.

Men who are incidentally diagnosed with PrCa will have T1a or T1b clinical stage cancer. As a result, those with Gleason grade 6 and PSA <10 ng/mL will by definition have NCCN low‐risk disease and be considered candidates for AS. However, here we have shown that men diagnosed with TURP have a higher risk of harboring aggressive disease compared with men who were diagnosed with needle biopsy, but have otherwise similar clinicopathologic and molecular prognostic features. This is not the first study to provide evidence that PrCa diagnosis by TURP is a poor prognostic indicator; Meachem et al. concluded that tumors causing obstructive voiding symptoms—a common reason for TURP—have poor prognosis.^22^ Another possibility is that the clinical stage, when applied to incidentally diagnosed tumors from TURP specimens (T1a or T1b), is poorly calibrated in risk models that are based primary on needle biopsies and, as a result, underestimate progression risk. Finally, the TURP procedure typically removes adenomatous tissue mostly from the transitional zone of the gland, whereas most prostate cancers arise from the posterior zone. While some tumors will arise from the transitional zone, an accurate determination of the total cancer volume and prostatic involvement by tumor from TURP is difficult to assess.

This study was retrospective, which can lead to unknown biases in patient selection. The TURP samples are from an archival cohort, so the treatment of these patients does not necessarily reflect modern practice. However, older cases are needed to obtain the extended follow‐up necessary to evaluate the association between molecular phenotypes and fatal outcomes. As a result of their age, a valid CCP score could not be generated in just over 10% of these samples. This is not expected for contemporary samples. Finally, many of these men had intermediate‐ or high‐risk disease and would not be considered candidates for conservative management based upon current guidelines.

The CCR score is prognostic and provides substantially more information about PCM than does a prognostic model based on clinicopathologic features alone. As such, CCR provides useful information and guidance for improving clinical management among men who are diagnosed with PrCa through TURP.

## CONFLICT OF INTEREST

S.S., L.L., D.D.F., S.R., and T.C. were employees of Myriad Genetics, Inc. at the time of this work and received salary and stock options as compensation. J.M.C. is a consultant for Myriad Genetics, Inc. H.M., D.M.B., and P.T.S. have nothing to disclose.

## AUTHOR CONTRIBUTIONS

All authors had full access to the data in the study and take responsibility for the integrity of the data and the accuracy of the data analysis. *Conceptualization*, J.M.C., S.S., H.M., D.M.B., P.T.S.; *Methodology*, F.M.L.; *Investigation*, J.M.C., S.S.; *Formal Analysis*, J.M.C., L.L., D.D.F., S.R.; *Resources*, H.M., D.M.B.; *Writing—Original Draft*, J.M.C., S.S., L.L., D.D.F., S.R., T.C.; *Writing—Review & Editing*, J.M.C., S.S., L.L., D.D.F., S.R.; *Visualization*, F.M.L.; *Supervision*, S.S., T.C., P.T.S.; *Funding Acquisition*, F.M.L.; *Data Curation*, J.M.C., L.L., D.D.F., S.R.; *Validation*, J.M.C.

## ETHICAL STATEMENT

National ethics approval was obtained from the Northern Multicenter Research Ethics Committee, followed by local ethics committee approval at each of the collaborating hospitals for the TURP cohort.

## Supporting information


**Figure S1**: Calibration plot comparing the predicted risk in quartiles modelled from the TURP1A cohort, and the observed risk from the TURP1B cohort. There is no evidence that the predicted risk is different from the observed risk (Greenwood‐Nam‐D'Agostino p‐value = 0.28).

## Data Availability

Requests to go to a review group for the study using the application for data form on the below link: https://www.qmul.ac.uk/wolfson/about-us/centres/ccp/data-sharing/.
