# Supplementary material for: Validation of the cell cycle progression score to differentiate indolent from aggressive prostate cancer in men diagnosed through transurethral resection of the prostate biopsy
Source: Cancer Rep (Hoboken). 2021 Aug 22;5(8):e1535. doi: 10.1002/cnr2.1535 (PMC9351676; doi:10.1002/cnr2.1535)

**Supplemental Material**

**Supplementary figure 1:** Calibration plot comparing the predicted risk in quartiles modelled from the TURP1A cohort, and the observed risk from the TURP1B cohort. There is no evidence that the predicted risk is different from the observed risk (Greenwood-Nam-D’Agostino p-value = 0.28).


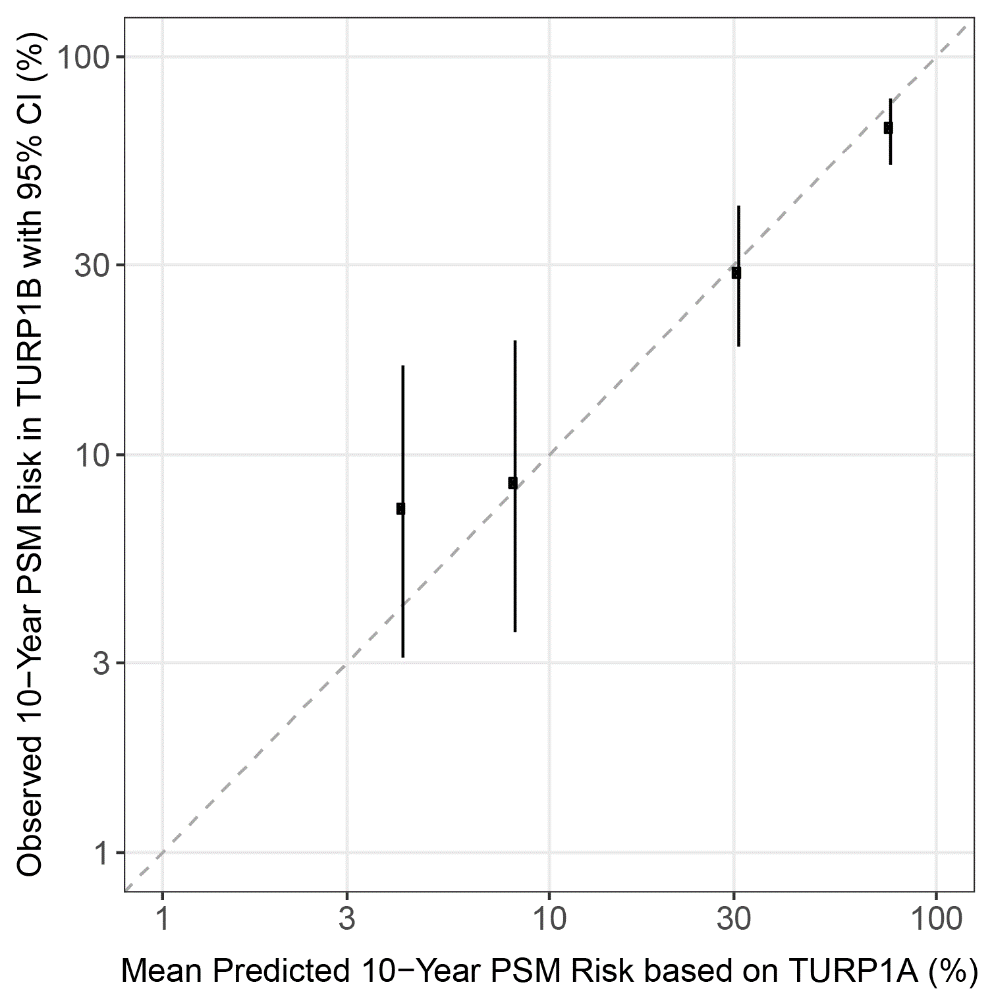

Supplement: Supplementary file 1 — Figure S1: Calibration plot comparing the predicted risk in quartiles modelled from the TURP1A cohort, and the observed risk from the TURP1B cohort. There is no evidence that the predicted risk is different from the observed risk (Greenwood‐Nam‐D'Agostino p‐value = 0.28). [file CNR2-5-e1535-s001.docx]
